# Solitary Primary Intracranial Extramedullary Plasmacytoma With Lymph Node Metastasis

**DOI:** 10.7759/cureus.23767

**Published:** 2022-04-02

**Authors:** Anna-Lena Meinhardt, Christopher W Sandifer, Manish Dave

**Affiliations:** 1 Internal Medicine, Rutgers University New Jersey Medical School, Newark, USA; 2 Hematology and Medical Oncology, Cooperman Barnabas Medical Center, Livingston, USA

**Keywords:** solitary extramedullary plasmacytoma, solitary bone plasmacytoma, hematologic malignancy, solitary intracranial plasmacytoma, multiple myeloma, intracranial plasmacytoma

## Abstract

Multiple myeloma is a neoplastic disease of plasma cells. Plasma cell disorders can present as a single lesion (solitary plasmacytoma) or as multiple lesions (multiple myeloma). Solitary plasmacytomas can occur in bone (plasmacytoma of bone) or in soft tissues (extramedullary plasmacytoma), and both can serve as a precursor lesion to multiple myeloma. These lesions may occur anywhere, however, intracranial presentations are rare. Here, we present a rare case of solitary intracranial extramedullary plasmacytoma in a patient complaining of headaches and vision changes. Despite radiation treatment, intracranial progression and rare lymph node involvement were seen soon after, prompting myeloma-directed therapy followed by autologous stem cell transplant, which have resulted in remission to date.

## Introduction

Solitary plasmacytoma lies within the spectrum of plasma cell disorders which include monoclonal gammopathy of undetermined significance (MGUS), polyneuropathy, organomegaly, endocrinopathy, monoclonal gammopathy, and skin changes (POEMS) syndrome, systemic light-chain (AL) amyloidosis, smoldering myeloma and multiple myeloma (the most common form). In rare cases, this proliferation occurs in the form of a solitary lesion, called a solitary plasmacytoma. These can present in the bone (solitary bone plasmacytoma {SBP}) or, less frequently, in soft tissue (solitary extramedullary plasmacytoma {SEP}), overall account for 3% of all plasma cell neoplasms and may develop in any tissue [1]. SEPs are more likely to develop locally whereas SBPs are more likely to progress to multiple myeloma over time [2]. There are a handful of case reports and case series documenting intracranial or intraparenchymal involvement of both SBPs and SEPs. In a large Mayo Clinic study, CNS involvement accounted for 0.7% of cases of plasma cell neoplasms, however, this more commonly occurs in the setting of progressive disease [3]. An extensive search was unable to find documented cases of primary extramedullary intracranial plasmacytomas with metastasis outside of the CNS. Treatment beyond radiation therapy in relapse/refractory cases is not well established and is based upon the best available evidence which includes case reports and expert opinion [4]. Here, we describe a patient with a history of vision changes and headaches who was found to have an intracranial plasmacytoma as the cause for her presenting symptoms, for which she received debulking surgery and radiation therapy. Relapsed disease was later discovered in a hypermetabolic axillary node.

## Case presentation

**Figure 1:**
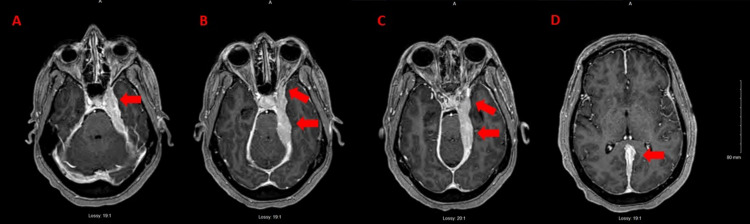
MRI sequences before biopsy of dural lesion. MRI post-axial T1 stealth sequence demonstrating enhancement of cavernous sinus (A), extension into the sella (A and B) and left optic nerve (B), enhancement of the tentorium (B and C), and extension into the falx (D).

A 54-year-old female presented to us with three weeks of left-sided headaches and vision changes. A non-contrast MRI demonstrated a 1.1 cm nodule in the left middle cranial fossa, which appeared to be a meningioma. The patient’s headaches persisted and a repeat MRI with contrast was done seven months later. This demonstrated an increase in irregular dural thickening and extension of abnormal enhancement into the adjacent cavernous sinus leading into the left orbital apex with rightward displacement of the sella along with prominent involvement of the tentorial leaflets and the falx. Diffuse parenchymal enhancement was also noted in the adjacent left (L) medial temporal lobe (Figures 1A-1D). A biopsy with debulking surgery of the lesions was performed and the intraoperative read noted dense chronic inflammation with the differential suggesting malignancy such as lymphoma. The dural biopsies along with biopsy of the left medial temporal lesion demonstrated infiltration by a dense lymphoplasmacytic population.

**Figure 2:**
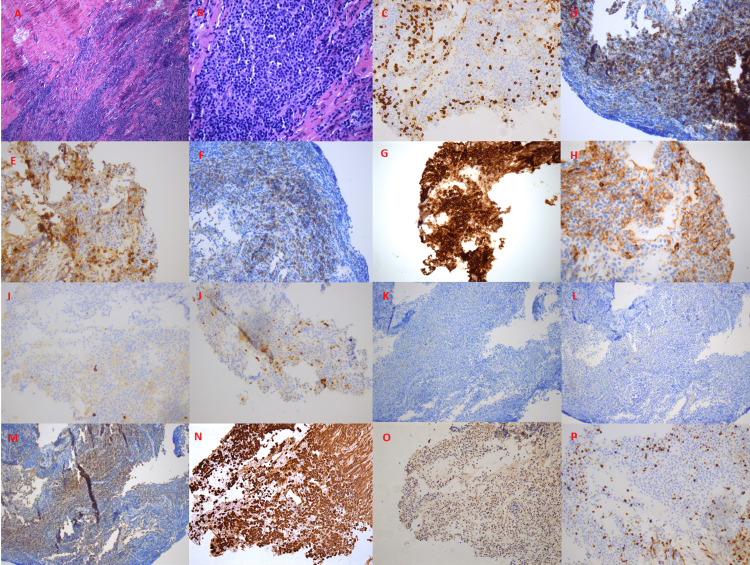
Dural biopsy of CNS lesion. Immunohistochemistry images are showing (A and B) dense infiltration by a lymphoplasmacytic cell population (H&E stain 100x and 400x, respectively), (C) CD3- stain, (D) CD19+ stain, and (E) CD20- stain. Though a fair amount of cells stained positive for CD20, these represent admixed B-cells unrelated to the clonal process that was shown to be polytypic by flow cytometry. (F) CD38+ stain, (G) CD138+ stain, (H) CD56+ stain, (I) CD117- stain, (J) cyclin D1- stain, (K) IgM- stain, (L) IgA- stain, (M) IgG+ stain, (N) kappa+, (O) lambda-, and (P) Ki67 15%. This is consistent with plasma cell dyscrasia with IgG kappa expression. CD: cluster of differentiation

Immunohistochemistry was performed to characterize the biopsies, which revealed a dense lymphoplasmacytic population consisting predominantly of cluster of differentiation (CD) 138-positive plasma cells, with focal dim expression of CD56, that were negative for CD117 and cyclin D1 with fewer CD20 positive small B-cells and CD3+ small T-cells. Ki67 demonstrated a proliferation index of ~15%, and plasma cells were shown to stain for kappa light chain and were negative for lambda chain (Figures 2A-2P). Flow cytometry revealed an abnormal plasma cell population accounting for 22.4% of analyzed events with a CD38+ (bright), CD19+, CD20-, CD56+, and intracytoplasmic kappa light chain restriction immunophenotype. Additionally, the sample contained a mixture of phenotypically unremarkable T-cells, polytypic B-cells, viable cells, and debris.

**Figure 3:**
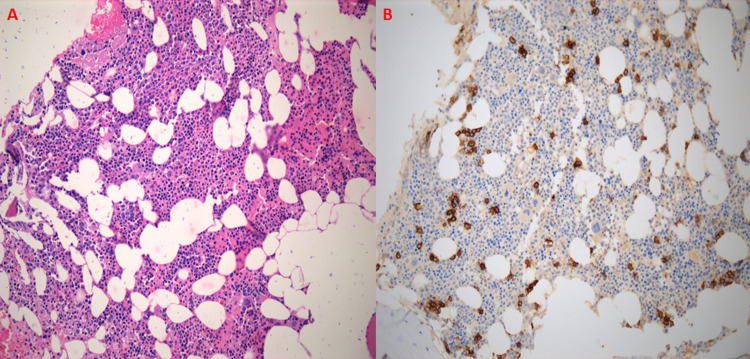
Bone marrow staging evaluation. (A) H&E shows 60-70% cellularity and (B) CD138+ cells demonstrate approximately 7% of marrow cellularity composed of plasma cells. The plasma cells were polytypic with respect to kappa and lambda (not available). CD: cluster of differentiation

Further workup included a bone single-photon emission computed tomography (SPECT) (showing no malignant disease in the bone), and an MRI of the thoracic spine was also unremarkable. SPEP and urine protein electrophoresis (UPEP) were unremarkable, and immunofixation showed two IgG kappa monoclonal protein bands. HIV and Epstein-Barr virus (EBV) testing were negative. Kappa/lambda ratio was elevated at 4.63. A bone marrow biopsy only showed mildly hypercellular bone marrow with 7% polytypic plasma cellularity (Figures 3A, 3B). FoundationOne Heme testing did not reveal any genomic alterations. The patient, therefore, received the rare diagnosis of a solitary extramedullary intracranial plasmacytoma.

**Figure 4:**
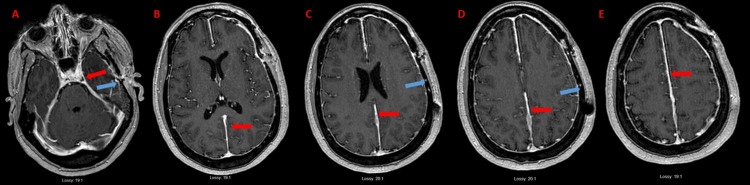
Follow-up MRI post-debulking surgery and RT. MRI post-T1 axial series show (A) enhancing tumor appears to have resolved at the sella, cavernous sinus, L optic nerve, and tentorium, (B-D) along the falx with no significant residual disease (red arrows), post-surgical changes (blue arrow). RT: radiation therapy; L: left

A positron emission tomography (PET)/CT revealed low-grade activity of standardized uptake value (SUV) 2.8 in an otherwise normal-appearing 1.0 cm left axillary lymph node, which was of uncertain significance. Physical examination and subsequent mammogram and breast ultrasound were normal, so no further intervention was performed. Based on this workup, there was no indication for systemic chemotherapy, so intensity-modulated radiation therapy (IMRT) was performed, delivering 4500 cGy to the tumor in 25 fractions. One month after completion, a repeat MRI seemed to show that the tumor had resolved with no significant residual disease (Figures 4A-4E).

**Figure 5:**
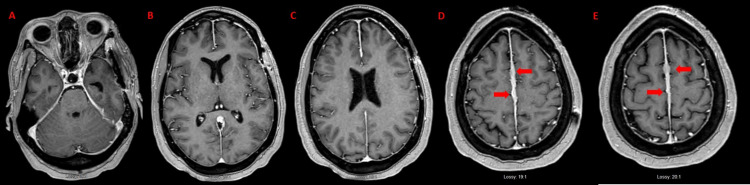
Six-month follow-up MRI post-debulking and RT. MRI post-T1 axial series show (A) overall stable disease at the sella, cavernous sinus, L optic nerve, tentorium, and (B and C) inferior falx. (D and E) Along the superior falx, particularly at the level of the frontal lobes, dural enhancement appears thicker, and more lumpy-bumpy compared to prior. This may signify slow-growing volume of extraaxial disease along the falx (red arrows). RT: radiation therapy; L: left

**Figure 6:**
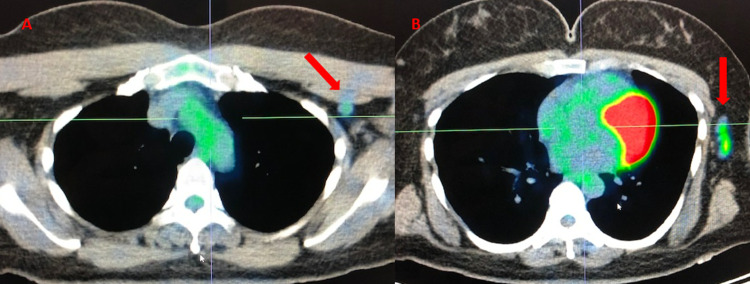
PET-CT six months after completion of debulking surgery and RT. PET-CT shows (A) hypermetabolic uptake in L subpectoral lymph node SUV 2.8 and (B) L axillary lymph nodes SUV 4.8 (red arrows). PET: positron emission tomography; RT: radiation therapy; L: left; SUV: standardized uptake value

**Figure 7:**
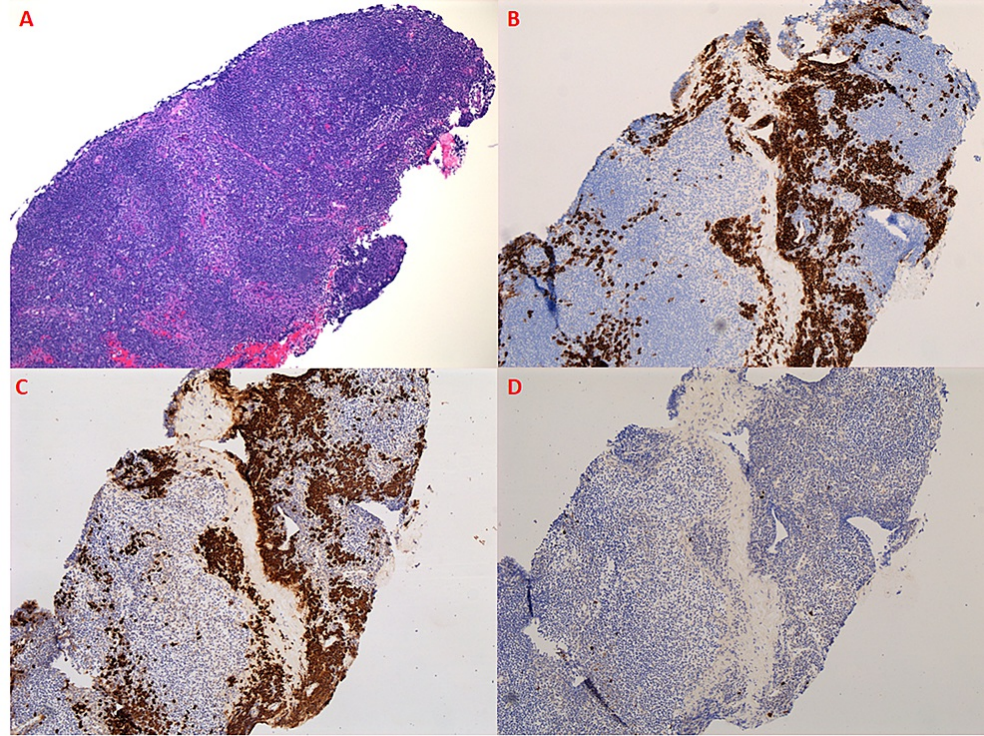
Hypermetabolic left axillary lymph node biopsy. The image shows (A) lymph node tissue with focal follicles and many interfollicular plasma cells with few admixed histiocytes (H&E 100x). Immunohistochemical stains (B: CD138, C: kappa IHC, and D: lambda IHC) show cells of the lymph node to be CD138+, positive for kappa, and negative for lambda. CD: cluster of differentiation; IHC: immunohistochemistry

Further repeat imaging six months after RT however revealed increased extra-axial/dural enhancement along the falx, suspicious for tumor progression, and a newly enlarged left-sided submandibular lymph node was detected clinically (Figures 5A-5E). Repeat bone marrow biopsy showed mild polytypic plasmacytosis (5%), K/L ratio increased to 14.36. PET/CT done at this time demonstrated continued hypermetabolic activity of the left axillary lymph node now increased to SUV 4.8 but with stable size of 1 cm (Figures 6A, 6B). At this time, it was decided to pursue biopsy. This showed CD138+, kappa+ plasma cells, and only few CD20+ and CD3+ cells in the background, consistent with a plasma cell neoplasm (Figures 7A-7D). Therefore, systemic therapy was begun with daratumumab, lenalidomide, bortezomib, and dexamethasone (D-RVd), with the plan for subsequent autologous stem cell transplant (ASCT).

**Figure 8:**
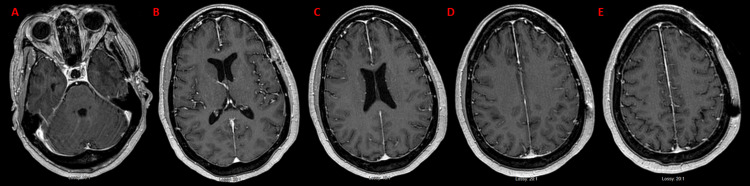
MRI brain after completion of four cycles of D-RVd. MRI post-T1 axial series show (A-E) no intra- or extraaxial masses are found. D-RVd: daratumumab, lenalidomide, bortezomib, dexamethasone

After completion of all four cycles, the free K/L ratio had decreased to 0.87. MRI showed resolution of disease along the falx compared to the prior image six months prior, and PET scan revealed only mildly avid axillary lymph nodes with no other hypermetabolic foci concerning malignancy (Figures 8A-8E). A repeat bone marrow biopsy showed no plasma cell involvement.

**Figure 9:**
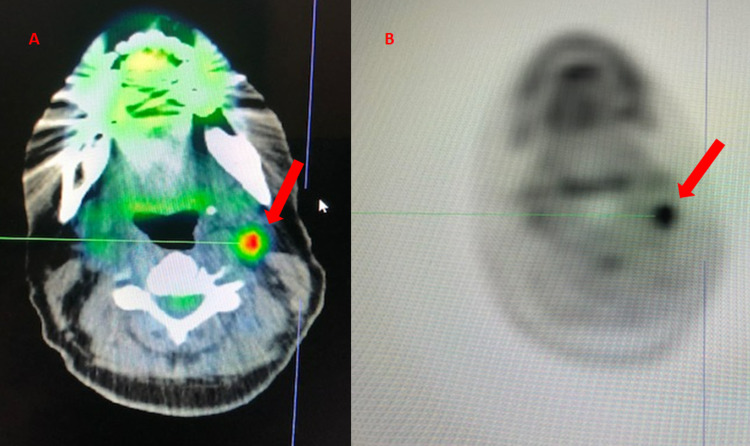
PET-CT one month after completion of ASCT. The images show (A and B) PET-CT completed one month after completion of ASCT demonstrating 18-FDG hypermetabolic uptake in level 2-3 left cervical lymph node less than 1 cm in size, SUV max 6.9. PET: positron emission tomography; ASCT: autologous stem cell transplant; FDG: fluorodeoxyglucose; SUV: standardized uptake value

The patient then underwent autologous stem cell transplant with a melphalan and thiotepa conditioning regimen. Approximately one month after her ASCT, a PET-CT was performed which demonstrated new fluorodeoxyglucose (FDG) avidity in the left cervical level 2-3 lymph nodes (Figures 9A, 9B). The patient then resumed her D-RVd for four months and had resolution of the metabolically active cervical lymph nodes, with a plan for maintenance therapy with D-Rd.

## Discussion

Solitary plasmacytomas have a yearly incidence of less than 500 cases in the United States, making it more difficult to study clinical courses or optimal treatment strategies [5]. These tumors are commonly found in the head and neck area, mainly in the upper aerodigestive tract, where they cause symptoms, such as nasal obstruction, epistaxis, and rhinorrhea [6]. Intracranial presentations however are rare and manifest with headaches, visual problems, seizures, or cranial nerve palsies [7]. The development of these tumors, like most other hematopoietic neoplasms in the CNS, has been much debated. Possible explanations include transformation of a peripheral B-cell with subsequent migration to the CNS, destruction of systemic tumor cells by the immune system with survival of cells that are hidden in the CNS [8], and specifically the deletion of p53 [9]. Even after imaging is obtained, they present a diagnostic challenge and are often misdiagnosed as other conditions. When there is no history of multiple myeloma, intracranial plasmacytomas are most commonly mistaken for meningiomas or subdural hematomas and biopsies are therefore necessary [10]. Diagnosis in our patient was slightly more challenging as well due to CD19 positivity, which can be seen occasionally in multiple myelomas, and according to one study was seen in a high fraction of extramedullary plasmacytomas [11]. In order to make a final diagnosis, multiple myeloma has to be excluded through bone marrow biopsy, blood work, and imaging, and the patient should be negative for CRAB criteria or any myeloma defining events. Especially in light of the need for tissue sampling in intracranial plasmacytomas, complete resection is nearly always the initial therapeutic approach. This is usually followed by adjuvant radiotherapy, as this is a highly radiosensitive neoplasm, and/or chemotherapy [5].

Of the two types of solitary plasmacytoma, SBP has been shown to progress to multiple myeloma (MM) at a significantly higher rate than SEP, with an estimated risk of progression as high as 25-30% in SEPs [12]. Interestingly, however, if SEPs do progress, they do so within three to five years of diagnosis while SBPs commonly also progress long after initial diagnosis [13]. SEPs may present in any organ and may metastasize locally or to distant lymph nodes or other sites. As these tumors are most commonly found in the upper aerodigestive tract, the most common site of metastasis is to the cervical lymph nodes, even after RT to the primary tumor [2]. Intracranial SEP has been reported to affect the meninges, the brain parenchyma, the skull base, or the ventricles. SEP may also rarely present as primary lymph node plasmacytomas, however, only a few cases have been documented. Overall survival once MM is diagnosed, is similar regardless of the original lesion [13]. More than one-third of all patients with solitary plasmacytoma progress to MM within three to five years, which is why close follow-up and identification of high-risk patients are key [13]. Aside from tumor size and osseous localization, increased light chains, and presence of paraprotein (both before and persistence after treatment) and anemia are also indicators for more rapid progression [12,13]. The presence of extramedullary intracranial disease has also been suggested to be a negative predictor of survival compared to patients with traditional multiple myeloma [14].

Standard treatment is commonly radiation and/or surgery based on location, but the use of adjuvant systemic therapy is still uncertain at this time, with studies showing either modest or no benefit. A recent retrospective study in France did show a benefit in progression-free survival without a benefit in overall survival when using combination therapy in the frontline setting, however, this was specific to SBP [15]. Once a patient has progressed to multiple myeloma, treatment should be myeloma specific. SEPs as in our patient are very rare and due to low case volume, there is no good consensus management beyond surgery and radiotherapy in the frontline setting, however, adjuvant chemotherapy may be considered in patients with tumors >5 cm and those with high-grade tumors [4]. In the relapsed/refractory setting, treating as multiple myeloma is indicated, however, both of these guidelines for use of adjuvant chemotherapy are of level IV evidence [4]. Relapse in our patient may be explained by the extent of involvement of plasmacytoma. RT may not have completely encompassed the full extent of the lesion resulting in tumor escape. Due to relapse, it was therefore decided to initiate Dara-RVd in our patient based on the GRIFFIN trial to ensure the deeper response observed in this trial prior to consolidation with ASCT, as well as due to the activity of the regimen in multiple myeloma, and the disease interrelatedness to plasmacytoma [16].

A small case series in Finland evaluated ASCT in high-risk plasmacytoma, which may show benefits in a subset of patients, however, there have yet to be any RCTs evaluating its use. Furthermore, the study included only two cases of extramedullary plasmacytoma [17]. Due to CNS presentation being extremely high risk, our patient additionally underwent bone marrow transplant per multiple myeloma treatment protocols with modification of the standard melphalan conditioning regimen. Thiotepa is known to have high penetration into the CNS and has been used successfully in the past [18,19]. Incorporation of thiotepa in this particular case was extrapolated from a recent study demonstrating favorable outcomes with thiotepa-containing regimens in primary CNS lymphoma which was presented at the American Society of Hematology (ASH) in 2020 [18,19]. Further clinical trials evaluating its use are still needed.

## Conclusions

Solitary intracranial plasmacytoma is a rare presentation and experiences are mainly documented in the form of case reports. This study yields less than a dozen reports of solitary intracranial plasmacytoma without the presence of multiple myeloma. Findings on imaging of the CNS tend to be nonspecific, which is why biopsy and histological analysis are critical.

Resecting as much of the tumor as possible has been shown to be critical for a patient’s outcome, however, this can prove to be challenging given the proximity to brain parenchyma, so post-operative radiation is crucial. Adjuvant chemotherapy does not yet have an established role in the prevention of progression but has been discussed in this study. Multiple myeloma-directed therapies should be implemented at relapse/progression, even if evidence is limited. There is currently a lack of guidance for optimal induction regimens, use of ASCT or conditioning regimens, or maintenance therapy in plasmacytoma therefore, determining the optimal treatment strategy beyond surgery/RT remains dependent on clinicians’ judgment and individual patient presentations in the highly unique setting of an intracranial presentation if refractory or at relapse.
